# New York Letter

**Published:** 1896-02

**Authors:** 


### NEW YORK LETTER.

NEW YORK, January 4, 1896.

The annual meeting of the Academy of Medicine for the election of officers, took place Thursday evening January 2, and the following were elected: for vice-president, Dr. Egbert H. Grandin; trustee, Dr. Joseph E. Janvrin; members of the committee on library, to serve four years—Dr. Kenan L. Collyer—to serve five years, Dr. B. Farquhar Curtis; member of committee on admissions, Dr. Robert A. Murray; delegate to the Medical Society of the State of New York, Dr. William S. Gottheil, Dr. Joseph Collins, Dr. R. H. Sayre, Dr. R. L. Parsons, Dr. C. H. Richardson. The election for president did not take place this year, and the present incumbent, Dr. Joseph D. Bryant holds over until next year. The reports of committees show that there were admitted forty-eight resident and thirteen nonresident fellows during the year and that twenty-two deaths occurred. The library committee reported that there were over 33,000 volumes in the library and over 600 medical journals were subscribed for. The committee was voted an appropriation of $4,500 for the year. At this meeting, a paper was read by Dr. F. H. Wiggin on "Infantile Intussusception," a study of 103 cases treated either by intestinal distention or laparotomy.

It has always seemed to me curious that any one could be induced to read a paper at the annual meeting of the Academy, for no matter how interesting the subject, there are but few listeners; there is a constant interruption from members entering and leaving the hall. Everybody has his ears open for the result of the election, and the paper receives scant attention, while outside the door, in the hall and polling room, the groups of doctors greeting each other and chatting far outnumber those within, forming a melancholy contrast, which once seen, would tend to deter one from caring to hold forth on that night.

A special meeting of the Academy was held on December 27 to take action on the following resolutions:

*Resolved*, That the New York Academy of Medicine deprecates the action of the commissioners of charities and corrections in abolishing the consulting board of Bellevue hospital and the consulting and visiting boards of the City, Harlem, Gouverneur, Fordham and Maternity hospitals, the Hospital for Nervous Diseases, the Almshouse, Workhouse, and Incurable hospitals and Randall's Island hospital.

*Resolved*, That the New York Academy of Medicine protests against the action of the commissioners of charities and corrections in placing the nominations, and, practically, the appointments in the hands of the incorporated medical schools and the fourth division of Bellevue (to all intents and purposes a monarchy), as contrary to the best interests of these institutions and of the medical profession.

*Resolved*, That a copy of these resolutions signed by the president and secretary of the New York Academy of Medicine be forwarded to his honor, the mayor, and to the commissioners of charities and corrections.

Unfortunately for the promoters of these resolutions, they have gone a little beyond the point at which they could have the sympathy of the profession at large. Resolutions condemning the high-handed method of dismissing medical men, without even acknowledgment of faithful services rendered, would doubtless have received a majority of the votes, but when the acceptance of the resolutions was put to a vote it was lost by an overwhelming majority.

*The Medical News* has changed its home, and will now be published in New York under the editorship, it is said, of Dr. J. Riddle Goffe, and Dr. George M. Gould, so long its able editor, will withdraw.

For a number of years every practicing physician in New York and vicinity has been annoyed in the extreme by receiving in his mail, once a month, at least, an envelope containing several cards giving name, address and office hours of a "specialist in female diseases." It is gratifying to learn that for a time, at least, they will be spared this nuisance, as the specialist in question, being under an indictment for an abortion, has disappeared and forfeited his bail.

December 29 was Hospital Sunday. According to the *Medical Record*, the amounts collected for the Hospital Association fund from the churches were: From the Protestant Episcopal, $16,117.61; Presbyterian, $1,688; Methodist,

$724.43; from the Synagogues $1,372.62; and Reformed, $959. All the returns are not in. Last year the association collected $58,208, of which $23,100 came from the churches.

On Mondays and Thursdays, at 5 P. M., from January 6 to February 20, the 19th annual course of clinical lectures on orthopædic surgery will be given at the Orthopædic Dispensary and Hospital in 59th street, free to physicians and medical studeents. The course is delivered by Dr. Newton M. Shaffer.

An interesting case was reported at the November meeting of the genito-urinary section of the Academy by Dr. R. Guiteras. It was a case of dislocation of the left testis from its position in the scrotum, upwards, and under the skin and dartos covering of the penis. It was of two months standing, the result of an accident. The patient, while riding on a heavily loaded wagon, attempted to jump from it, and fell under the wheel, which passed between his legs up over the left pubic bone and left side of abdomen. He was carried to a hospital where he was tended for three weeks and then returned home. He was, when seen by Dr. Guiteras, in good general condition but had some pain in left hip and pain and discomfort in the penis. Dr. Guiteras operated to replace the testis, making an incision over it along the left side of the penis back to the peno-scrotal junction, and a vertical incision in the scrotum down to and into the tunica vaginatis, which he had to stretch with the fingers before he could replace the organ. It had been torn away from the globus minor, which was still in place. The wound was then sutured throughout. After the operation, the patient experienced immediate relief from the discomfort in this region.

At the November general meeting at the Academy of Medicine, November 21, the anniversary discourse was delivered by Dr. E. G. Janeway on "Progress in Medicine." The paper was published in the New York *Medical Record* for December 7.

